# Connective tissue nevus—Isolated collagenoma

**DOI:** 10.1111/srt.13210

**Published:** 2022-10-25

**Authors:** Qingqing Yang, Shengxiu Liu

**Affiliations:** ^1^ Department of Dermatology The First Affiliated Hospital of Anhui Medical University Hefei Anhui China; ^2^ Key Laboratory of Dermatology, Anhui Medical University, Ministry of Education Hefei Anhui China; ^3^ Inflammation and Immune‐Mediated Diseases Laboratory of Anhui Province Hefei China

Dear editor

An 8‐years‐old boy presented with a single skin‐colored plaque the size of a pea on the left lumbar rib cage 2 weeks after birth. He came to the dermatology department because the rash had grown to the size of a palm with age. None had a family history, no history of trauma and medication. Dermatologic examination: skin‐colored plaque on the left side of the lumbar rib cage, measuring about 11 cm × 13.5 cm, with a rough surface and several subcutaneous plaques of different sizes distributed in local clusters, partially fused, soft and without pressure pain (Figure 1A). Reflective confocal microscopy showed mild epidermal thickening in the lesion area, with approximately normal intraepidermal structures and subdermal fibrous tissue hyperplasia (Figure 1B). Dermatoscopy showed scattered brown pigmented spots and pigmented network with unclear borders visible under the microscope (Figure 1C). Histopathology showed no abnormality in the epidermis and increased, thickened, and disorganized collagen fiber bundles in the dermis (Figure 2A). Masson trichrome staining showed a significant increase in blue collagen fibers (Figure 2B). Elastic staining showed a significant decreased in elastic fibers (Figure 2C). Diagnosis is isolated collagenoma (IC). The family refused surgical treatment.

**FIGURE 1:**
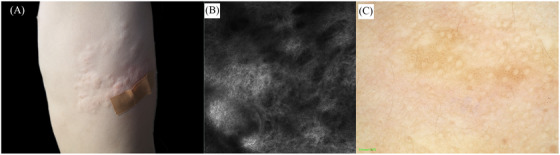
(A) General photo showed: skin‐colored plaque on the left side of the lumbar rib cage, measuring about 11 cm × 13.5 cm, with a rough surface and several subcutaneous plaques of different sizes distributed in local clusters, partially fused, soft and without pressure pain. (B) Reflective confocal microscopy showed: mild epidermal thickening in the lesion area, with approximately normal intraepidermal structures and subdermal fibrous tissue hyperplasia. (C) Dermatoscopy showed: scattered brown pigmented spots and pigmented network with unclear borders were visible under the microscope

**FIGURE 2:**
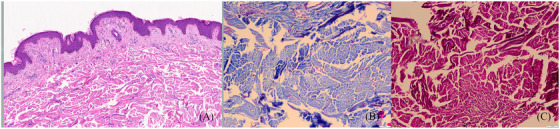
(A) Histopathological examination (HE staining) revealed: the epidermis was normal, and the dermis had an increase in haphazardly arranged collagen bundles. (B) Masson's trichrome staining showed a significant increase in blue‐colored collagen bundles (original magnification: ×200). (C) Elastic fiber staining showed diminished elastic fibers (original magnification: ×200).

## DISCUSSION

Connective Tissue Nevi (CTN) is a type of cutaneous malformation caused by an increase or structural alteration of the extracellular matrix components of the dermis, the main components of which are collagen, elastin and proteoglycans. The classification method proposed by Uitto et al.^1^ is currently used to classify CTN into five categories: collagenous, elastin, proteoglycans, mixed and fibroblast types, the first three of which can all manifest as hereditary and acquired. Among them, IC is a rare type of acquired collagen CTN with an unknown etiology, which has been suggested to be related to a limited proliferation of type I collagen and a reduced amount of collagenase.^2^ Nasrin Saki et al.^3^ have hypothesized that IC appearing in areas of friction on the body are associated with localized repetitive rubbing. It is characterized by asymptomatic papules, nodules and plaques that are solitary or confined to a single site with reduced pigmentation. The diagnosis of collagenoma requires, in addition to hematoxylin‐eosin staining to observe the histological changes, the genetic history, lesion characteristics and special histochemical staining to highlight the composition of CTN for differentiation. Yeser Genc et al.^4^ verified the histopathological features of typical dermatofibroma and atypical dermatofibroma by dermatoscopy, which provided a basis for the need of histopathology for clinical atypical dermatofibroma. In addition, reflective confocal microscopy is widely used in the diagnosis and treatment of skin tumors and inflammatory diseases.^5^ Therefore, in this case, for the first time, adjuvant techniques such as dermatoscopy and reflective confocal microscopy were applied to IC, and the results were consistent with the histopathological presentation.

The author analyzed and summarized it according to clinical manifestations combined with histopathological and skin imaging manifestations, aiming to provide clinicians with diagnostic reference.

## FUNDING

Key Research and Development Program of Anhui Province (202004j07020034).

## Data Availability

Data sharing not applicable to this article as no datasets were generated or analyzed during the current study
